# Comparison of methods for determining biogeochemical fluxes from a restored oyster reef

**DOI:** 10.1371/journal.pone.0209799

**Published:** 2018-12-26

**Authors:** Melanie Jackson, Michael S. Owens, Jeffrey C. Cornwell, M. Lisa Kellogg

**Affiliations:** 1 University of Maryland Center for Environmental Science, Horn Point Laboratory, Cambridge, Maryland, United States of America; 2 Virginia Institute of Marine Science, College of William & Mary, Gloucester Point, Virginia, United States of America; University of Saint Andrews, UNITED KINGDOM

## Abstract

Oyster reef restoration can significantly increase benthic denitrification rates. Methods applied to measure nutrient fluxes and denitrification from oyster reefs in previous studies include incubations of sediment cores collected adjacent to oyster clumps, benthic chambers filled with intact reef segments that have undergone *in situ* equilibration and *ex situ* incubation, and cores with single oysters. However, fluxes of nutrients vary by orders of magnitude among oyster reefs and methods. This study compares two methods of measuring nutrient and metabolic fluxes on restored oyster reefs: incubations including intact segments of oyster reef and incubations containing oyster clumps without underlying sediments. Fluxes of oxygen (O_2_)_,_ dissolved inorganic carbon (DIC), ammonium (NH_4_^+^), combined nitrate and nitrite (NO_2/3_^-^), di-nitrogen (N_2_), and soluble reactive phosphorus (SRP) were determined in June and August in Harris Creek, a tributary of the Chesapeake Bay, Maryland, USA. Regression of fluxes measured from clumps alone against those measured from intact reef segments showed significant positive relationships for O_2_, DIC, NH_4_^+^, and SRP (R^2^ = 0.920, 0.61, 0.26, and 0.52, respectively). Regression of clump fluxes against the oyster tissue biomass indicates significant positive relationships for O_2_ and NH_4_^+^, marginally significant and positive relationships for DIC and N_2_, and no significant relationship for NO_2/3_^-^ or SRP. Although these results demonstrate that the incubation of oyster clumps without underlying sediments does not accurately represent biogeochemical fluxes measured from the whole oyster and sediment community, this work supports the need to understand the balance between the metabolism of oysters and local sediments to correctly estimate biogeochemical rates.

## Introduction

In Chesapeake Bay, populations of the native oyster *Crassostrea virginica* have experienced substantial decline due to overfishing, disease, and habitat loss [1]. Oyster restoration has been applied as a management strategy with the dual goals of rebuilding the commercial fishery and restoring ecosystem services and benthic habitats [2, 3]. Oyster-associated ecosystem services that enhance nitrogen (N) removal are being considered for approval as best management practices (BMPs) in the Chesapeake Bay [4] where nitrogen inputs are a primary cause of eutrophication [5, 6]. Chronic eutrophication from N sources, such as septic systems and fertilizer runoff, can result in low oxygen “dead zones”, loss of seagrasses, and expansion of harmful algal blooms [7–9]. Oysters can remove N if filtration of particulate N [10] is followed by assimilation and sequestration of nutrients into tissues and shells, deep burial of nutrients in sediments, or enhancement of denitrification [11].

Oysters and other reef organisms appear to create optimal conditions for N removal via denitrification. Oysters remove oxygen through respiration [12], re-oxidation of reduced N, S and Fe, and biodeposit remineralization [13], which creates anaerobic environments for denitrifiers to reduce oxidized forms of N (NO_2/3_^-^) to N_2_ gas. Although the remineralization of biodeposits can result in fluxes of NH_4_^+^ from reefs [11,14], this form of N may become available to denitrification by coupled nitrification- the conversion of NH_4_^+^ to NO_3_^-^. Under aerobic conditions, attenuation of NH_4_^+^ concentrations via nitrification is associated both with living oysters and oysters shell [15] and with surfaces of other members of the macrofaunal community (i.e., polychaetes and amphipods) and bivalve soft tissues [16]. Given that reefs can provide as much as 50 m^2^ of surface area per square meter of reef [17], it is likely that they promote nitrification by providing an abundance of oxic surfaces on which they can grow. Despite this knowledge, the mechanisms that control this coupled process and eventual nitrogen removal by denitrification remain poorly understood.

Although observations show that denitrification associated with oysters effectively removes nitrogen [18–21], methodologies and results vary widely. Methods used to estimate denitrification and nutrient cycling associated with oysters include: benthic tunnels [14], sediment core incubations with the addition of oyster biodeposits [22, 23], incubations of sediment cores collected adjacent to oyster reefs [18, 24–28], incubations of live oysters without substrate [15], *in situ* experimental chambers that encompass intact reef segments [20], and *in situ* equilibration of intact reef segments with *ex situ* incubation and measurement [19]. Many of these studies do not include oysters or the highly abundant reef-associated organisms that alter biogeochemical cycles [18, 29]. Although progress has been made in developing methods for measuring nutrient fluxes from oyster reefs, less progress has been made in directly comparing flux measurements from the entire reef (acclimated oyster clumps plus sediment) to oysters alone. Oysters alone, here called “oyster clumps”, refer to live juvenile and adult oysters attached to shell or each other along with the associated reef community. Clumps typically had barnacles, mussels, tunicates and other sessile filter-feeding organisms attached and included small motile organisms such as polychaete worms, amphipods and mud crabs. The relative importance of oysters clumps in stimulating total reef denitrification remains a critical gap in our knowledge.

Much of the work on oyster-associated denitrification focuses on the filtration of organic matter from the water column and its deposition on sediments as feces and/or pseudofeces (i.e., biodeposits; [22]). Biodeposits concentrate organic matter on the aerobic sediment surface where the supply of labile carbon is expected to stimulate conversion of oxidized forms of N (NO _2/3_^-^) to N_2_ gas by denitrifying bacteria [11, 30–32]. In addition to oysters, restored reefs provide habitat for other filter-feeding organisms (e.g. mussels, tunicates, and barnacles) as well as deposit-feeding and bioturbating organisms, all of which can enhance N removal [19, 29, 33].

Contrary to studies that have focused on the impact of biodeposits on sediment denitrification, recent literature suggests live oysters are denitrification hot spots [15, 19, 21, 24]. Incubations of intact segments of oyster reef have produced some of the highest rates of denitrification, which can be attributed to the filtration of organic matter from the water column by oysters and reef-associated filter-feeders [19]. Incubations comparing live oysters to bare sediment have shown that live oysters have higher rates of denitrification [24], whereas incubations of shell have shown either lower or similar rates to live oysters [15, 21]. While experimental separation of oyster reef components helps locate where denitrification occurs, extrapolation from these studies to entire reef systems is problematic without the inclusion of associated reef organisms and underlying sediments. In addition, microbiome structures from live and dead shell, sediment, and oyster digestive glands suggest that the transformation of NO_2_^-^ to N_2_ is not controlled by the abundance of complete denitrifiers in the sediment but rather by complex interactions within the microbial community [21]. None of these previous studies have directly resolved the relative importance of oyster clumps in determining denitrification rates measured from entire restored reefs.

We combined field sampling and replicated laboratory flux chamber incubations with and without sediments to assess whether the denitrification and nutrient fluxes occurring on oyster reefs are associated with oyster clumps. We hypothesized that oysters alone would provide accurate estimates of denitrification compared to those measured from reef segments containing oysters and sediment, whereas oxygen demand and inorganic nitrogen dynamics (i.e. NO_2/3_^-^ and NH_4_^+^) measured from oysters alone may differ from whole reef incubations, due to the exposure of new surface area and absence of sediment microbial communities. This prediction was made following knowledge that denitrification was consistently higher from restored reef sites compared to control sediments [19], with the expectation that samples with higher oyster clump biomass densities would have higher biogeochemical rates.

## Materials and methods

### Study area and field sampling

**Fig 1:**
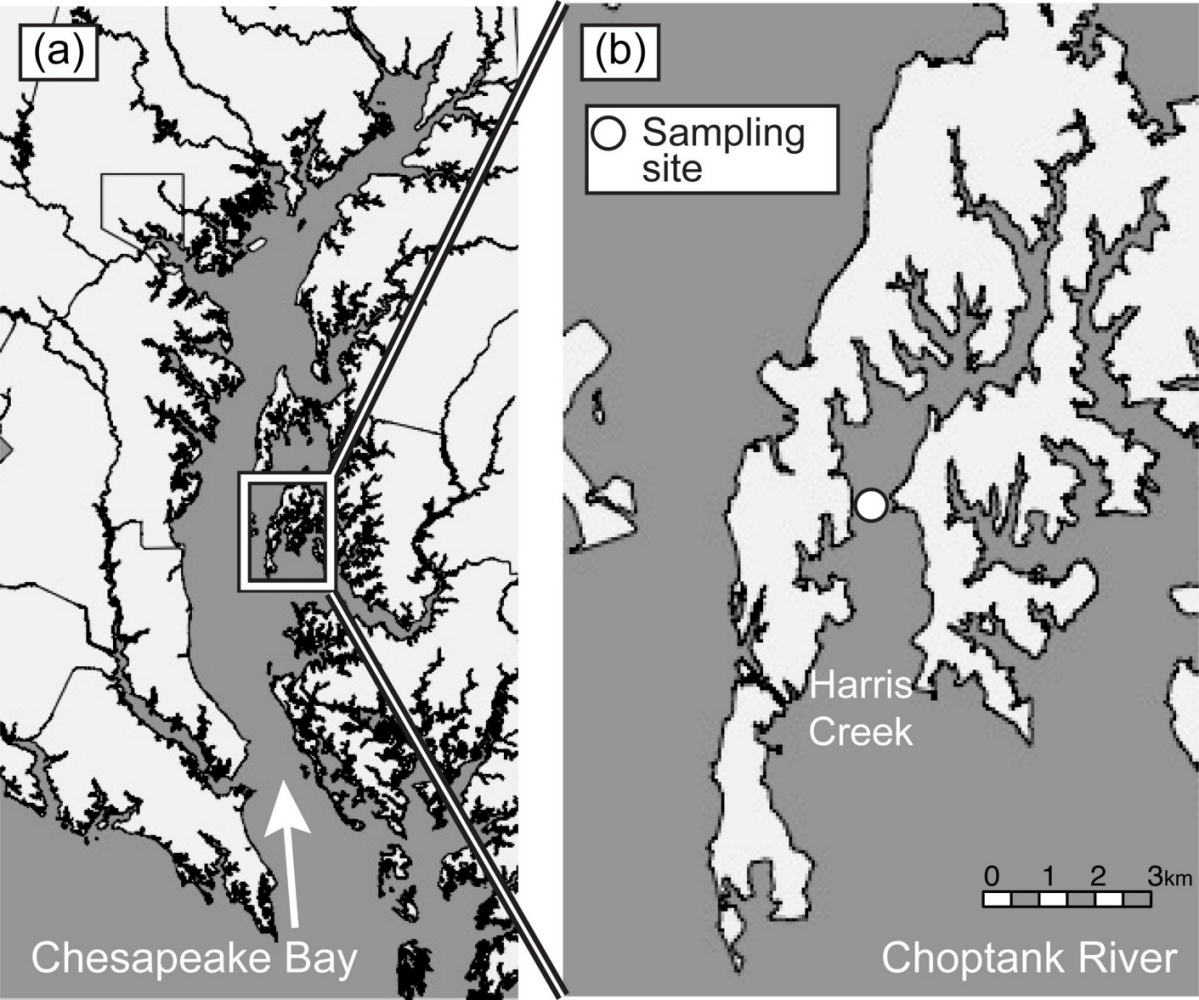
Map of study area. Map showing (a) the Choptank River a major tributary to the Chesapeake Bay and (b) the location of the study site (**○**) within Harris Creek (Map was created in R).

A restored reef in Harris Creek (38°45.129’ N, 76°18.571’ W), a tributary to the Choptank River, Maryland, USA (Fig 1) was chosen for the study area. The reef was restored by placement of 28.19 million juvenile oysters set on shell on 6.53 ha of suitable substratum in 2012 [34]. In 2015, the density of live oysters across the entire reef averaged 20.39 oysters per m^-2^ [34].

**Fig 2:**
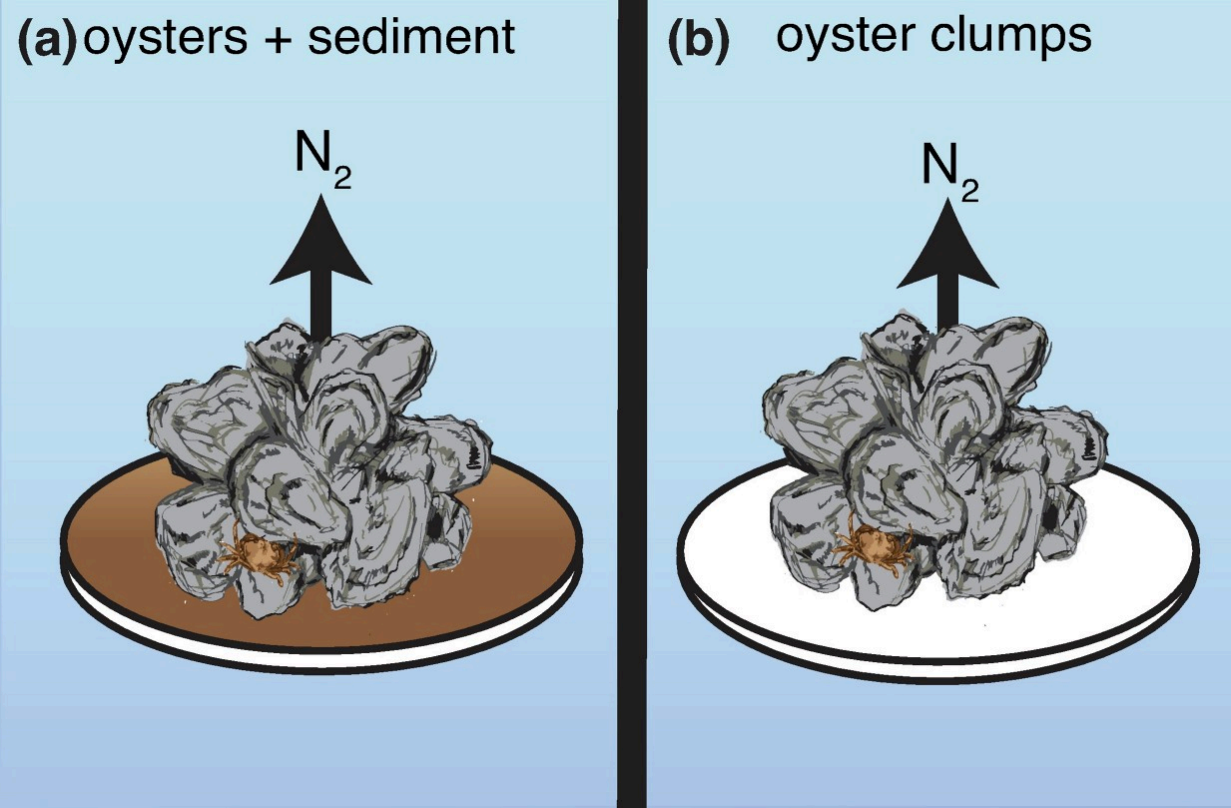
Diagram of incubation chamber setup. Diagram of (a) acclimated reef segments (oysters and sediment) for dark and light incubations and (b) oyster clumps alone for dark incubations.

Experiments were conducted in early and late summer (June and August) 2017 using the *in situ* equilibration of intact segments of oyster reef (hereafter “reef segments”) with *ex situ* incubation approach [19]. This work is a part of a larger ongoing project using equilibrated segments of oyster reef to understand spatial and seasonal patterns of nutrient fluxes in Harris Creek; however, the focus of this work is to determine the importance of oysters relative to intact reef segments. At least one month prior to incubations, divers embedded base trays filled with sediment, oyster shell, and live oysters in the substratum flush with surrounding sediments. Sediments consisted of fine-grained muds interspersed with shell fragments, also termed “shell hash”. Oyster biomass densities in trays ranged from 57 to 574 g DW m^-2^. After trays had equilibrated in the field for at least one month, divers capped the base trays with a pvc pipe midsection fitted with a lid and intact reef segments were returned to Horn Point Laboratory, Cambridge, MD where reef segments were placed in filtered Choptank River water with the temperature adjusted to Harris Creek conditions (23.3°C in June to 28°C in August). An empty tray filled with only filtered river water served as a “blank”. Prior to each set of incubations, trays were aerated for ~1 h to bring oxygen concentration to saturation. At the start of each incubation, chambers were capped with a stirring lid containing ports for sample collection, but sealed the sample from the surrounding water bath and stirred the water column with a motor-driven impeller [19]. As part of the larger project, reef segments were incubated first in the dark, then with illumination. After these incubations were complete, a subset of these samples (4 samples in June and 6 samples in August) was selected for additional study based upon whether the sample had at least one oyster over ~75 mm visible on the surface sediment. For each tray selected, the live oysters and oyster clumps were carefully removed from each tray, placed in clean and empty incubation chambers, aerated for ~1 h, and incubated in the dark (Fig 2). Our study focused on comparing the results from these “clump only” incubations to results from the dark incubations of the 10 intact reef segments from which they were removed. Fluxes for reef segments not selected for clump only incubations because oysters were too small or absent are not presented as part of the present study.

### Oyster abundance and biomass

Once incubations were complete, samples were rinsed over a 12.5-mm mesh sieve and all material ≥12.5 mm was frozen for later analyses. For each sample, all live oysters were counted and measured to the nearest mm. Shell lengths were converted to grams of oyster tissue dry weight from seasonal length to biomass regressions based upon samples collected from Harris Creek, MD 2015 (Kellogg et al. unpubl. data). The area of an individual tray (0.1 m^2^) was then used to calculate the oyster biomass density (g DW m^-2^).

### Biogeochemical flux measurement

Solutes (NH_4_^+^, NO_2/3_^-^, SRP) and dissolved gases (O_2_, N_2_, Ar, DIC = [H_2_CO_3_]+[HCO_3_^-^]+[CO_3_^2-^]) were collected approximately every 45 minutes (4 times total) during each set of incubations from a sampling tube fitted in the lid, while a water replacement tube pulled water from the water bath. Sampling intervals were determined based upon changes in dissolved oxygen (FireSting O_2_-Mini oxygen meter), and incubations of reef segments and oyster clumps alone had the same incubation duration. Final oxygen concentrations for reef segments and oyster clumps ranged from 0.14 ± 0.06 mmol l^-1^ (mean ≥ SD) in June to 0.15 ± 0.04 mmol l^-1^ in August. Dissolved gases were preserved with 10 μl of 50% saturated HgCl_2_, tightly sealed, submerged in water, and held at or slightly below incubation temperature until analysis. Solute samples were filtered through a 0.45 μm pore-size filter and kept frozen for analysis. N_2_, O_2_, and Ar concentrations were measured on a membrane-inlet mass spectrometer [35] within 2 weeks of collection. DIC concentrations were measured using an infrared-based analyzer (Apollo SciTech; [36]) with a calibration coefficient of variation that ranged from 0.03 to 0.06% with an average standard deviation of ≥6.09 μmol l^-1^. Phenol/hypochlorite colorimetry was used to determine NH_4_^+^ concentrations [37]. NO_2/3_^-^ was analyzed spectrophotometrically following Doane and Howarth [38], while a composite reagent of molybdic acid, ascorbic acid, and trivalent antimony were used to determine SRP concentrations [37]. Concentrations of gases and nutrients were regressed versus time to obtain a slope. Fluxes were considered significant if they had an R^2^≥0.80 and the change in concentrations over the incubation were greater than the precision of the analyses [39]. When it was significant, the slope of the “blank” was subtracted from the slope of sample fluxes to remove the effects of water column processes. Areal rates were determined based on the equation:

$$F=\frac{\Delta C}{\Delta t}*\frac{V}{A}$$

where F is the flux (mmol m^-2^ hr^-1^), ΔC/Δt is the slope (mmol L^-1^ hr^-1^), V is the volume of overlying water (L), and A is the area of the incubated tray segment (m^-2^). The overlying water volume was determined by subtracting the volume of sediment measured in the tray and the displacement volume of the live oysters from the total incubation chamber volume.

### Statistical analyses

We used a series of criteria to determine whether clump fluxes were representative of reef segment fluxes at this specific site. If the clump fluxes accurately estimate reef segment biogeochemistry, we would expect the two approaches to be highly correlated (R^2^≥0.80), have correlation coefficients that did not differ significantly from one, and have intercepts that did not differ significantly from zero. All statistical analyses were performed in R 3.1 using its “base” and “stats” packages [40]. Linear regression was used to determine the slope and whether there were significant linear relationships between fluxes measured from clumps without sediments and fluxes measured from reef segments. For fluxes that had a significant positive relationship, we used a two-tailed t-test of slope to determine whether the regression coefficient was significantly different from the expected slope of one. If the results from the incubation of oysters alone were an accurate estimate of intact reef segments, the correlation coefficient would be one. When slopes did not differ significantly from one, we used a linear model function to determine whether the intercept of the regression line was significantly different from zero. In addition, we used linear regression to test for relationships between biogeochemical fluxes and oyster biomass. Significance level for statistical tests was set at α = 0.05. P-values between 0.05 and 0.10 were considered marginally significant.

## Results

### Biogeochemical fluxes

**Fig 3:**
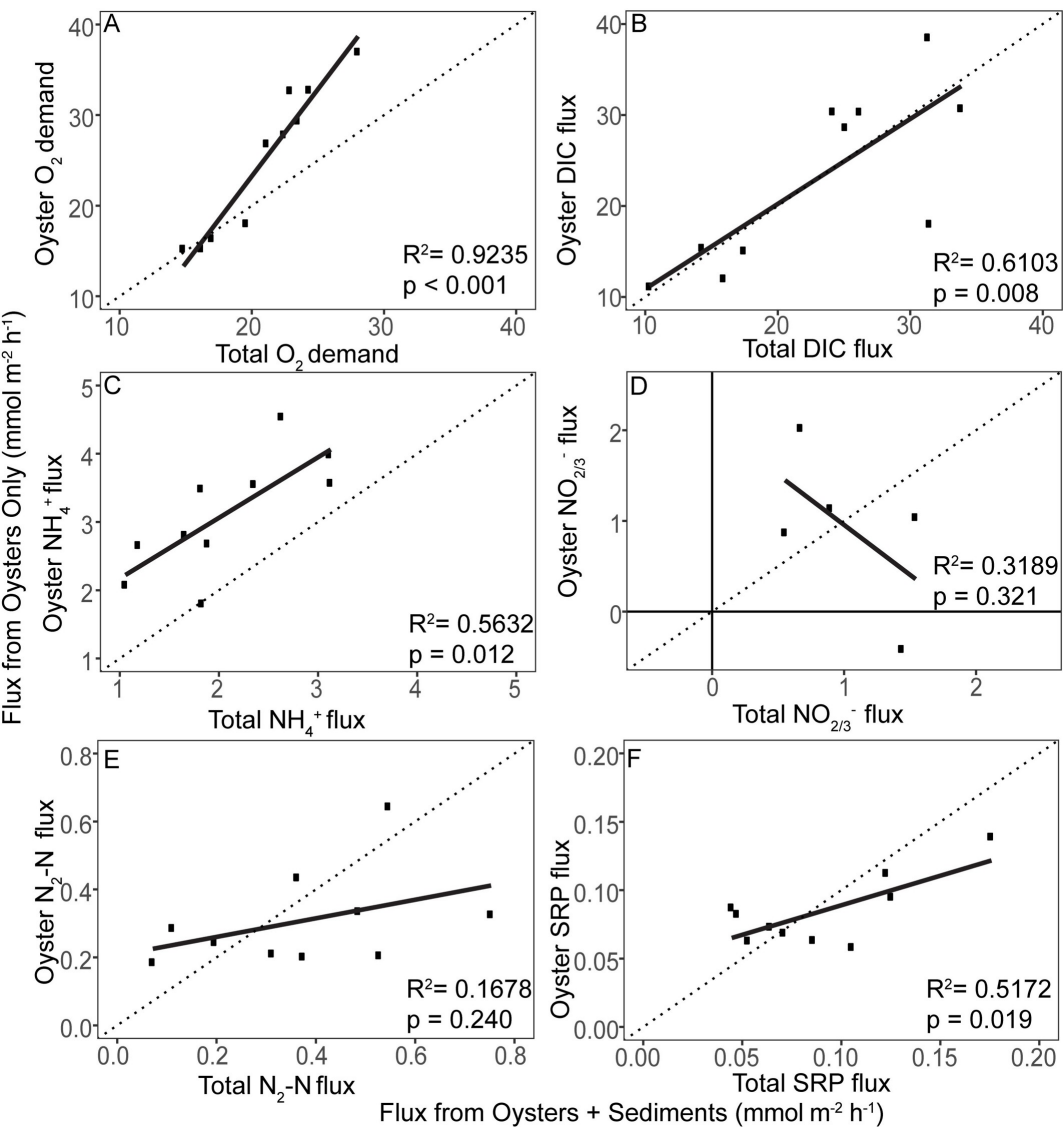
Regressions of fluxes from oyster clumps against those from reef segments. Regressions of (a) oxygen demand (b) DIC (c) NH_4_^+^ (d) NO_2/3_^-^ (e) N_2_ (f) and SRP fluxes.

**Fig 4:**
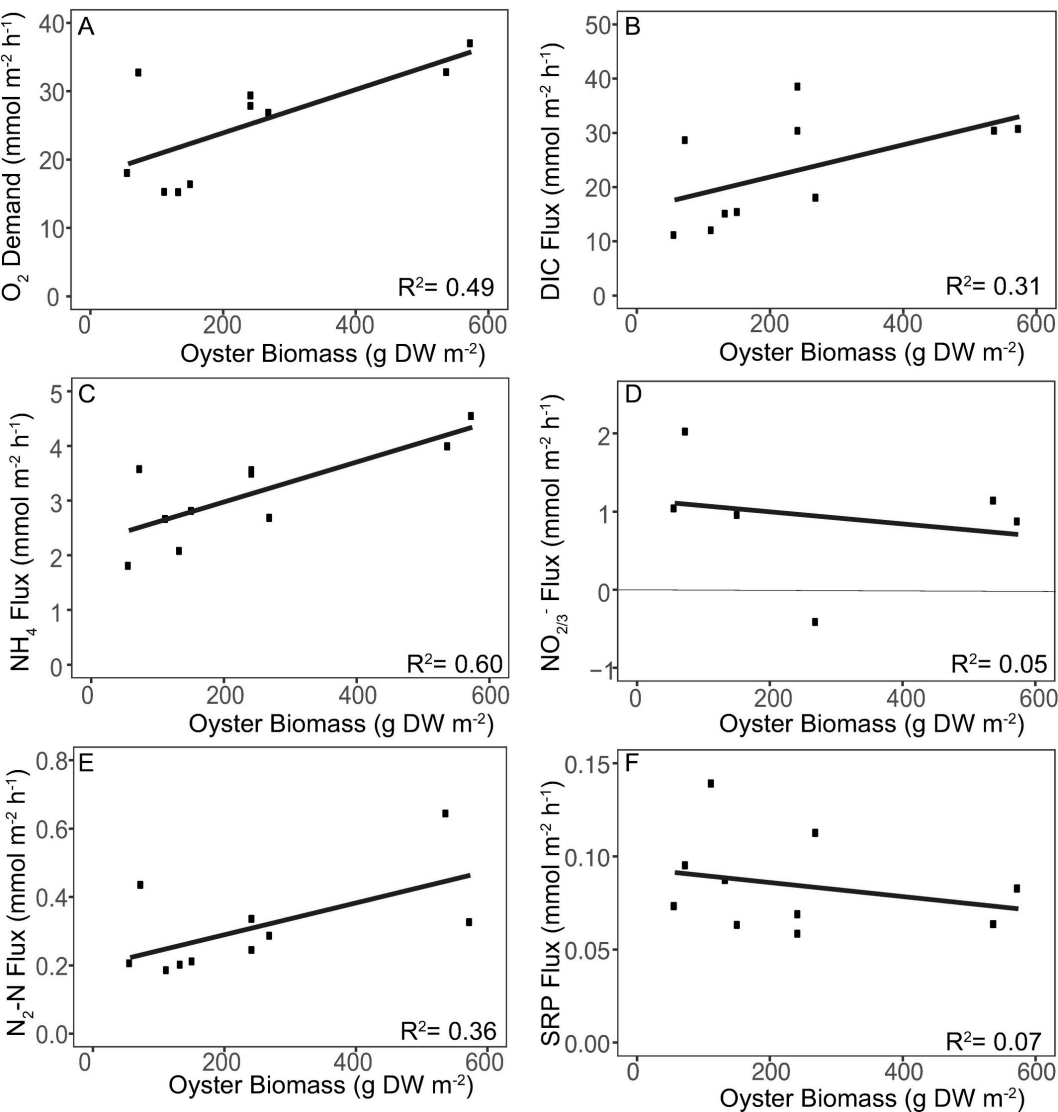
Regressions of fluxes from oyster clumps against oyster biomass. Regressions of (a) oxygen demand (b) DIC (c) NH_4_^+^ (d) NO_2/3_^-^ (e) N_2_ (f) and SRP fluxes.

The majority of fluxes for clumps alone showed positive relationships with fluxes from reef segments (Fig 3). Overall, fluxes from clumps alone increased with increasing biomass density (Fig 4).

Regression of oxygen fluxes from clumps against those from reef segments (Fig 3A) demonstrated a significant (p < 0.001), highly correlated (R^2^ = 0.92) positive relationship. However, the regression coefficient from the comparison of both methods was significantly greater than the predicted slope of one (*t* = -4.71, p = 1.5x10^-3^) with clumps generally overestimating oxygen demand, especially at high flux rates. The relationship between clump oxygen demand and oyster clump biomass density was significant and positive (p = 0.02, R^2^ = 0.49; Fig 4A).

DIC-based respiration rates did not differ significantly between the two incubation methods. Regression analyses (Fig 3B) demonstrated a significant positive relationship between clump and reef segment fluxes (p = 0.008) but were not as highly correlated (R^2^ = 0.61) as oxygen-based respiration rates. The relationship between the two methods was not significantly different from the predicted slope of one (*t* = 0.24, p = 0.81) and the intercept was not significantly different from the predicted value of zero (*t* = 0.23, p = 0.82). DIC fluxes tended to increase with oyster biomass, but were only marginally significant (p = 0.09, R^2^ = 0.31; Fig 4B).

Regression of NH_4_^+^ fluxes from clumps against those from reef segments (Fig 3C) demonstrated a significant (p = 0.012) positive relationship with correlation similar to that for DIC fluxes (R^2^ = 0.56). In addition, the regression coefficient from the measured relationship was not significantly different from the expected relationship (*t* = 0.41, p = 0.69) and the intercept was not significantly different from zero (*t* = 2.13, p = 0.07). However, NH_4_^+^ fluxes measured from oyster clumps consistently overestimated rates measured from reef segments. NH_4_^+^ fluxes from oyster clumps alone had a significant positive relationship with oyster clump biomass (p = 0.008, R^2^ = 0.60; Fig 4C).

Combined nitrate and nitrite concentrations were variable within the time course of experiments resulting in few regressions of concentration against time indicative of significant fluxes (R^2^ > 0.080). For the five clump fluxes that were significant, regression analyses found no relationship (p = 0.32, R^2^ = 0.321) between NO_2/3_^-^ fluxes from clumps versus those measured from reef segments (Fig 3D) and showed a trend towards a negative relationship between fluxes using the two different measurement approaches. Likewise, NO_2/3_^-^ fluxes were not related to oyster clump biomass (p = 0.67, R^2^ = 0.05; Fig 4D).

Rates of denitrification measured from clumps alone did not have a significant relationship (p = 0.24, R^2^ = 0.16; Fig 3E) to rates measured from reef segments but did show a slight tendency for the relationship to be positive, with denitrification rates from oyster clumps overestimated at the lower end of reef segment measurements and underestimated at the higher end. Regression of clump denitrification rates indicated a marginally significant positive relationship with oyster clump biomass (p = 0.07, R^2^ = 0.36; Fig 4E).

Regression analyses found a significant positive relationship between SRP fluxes measured from clumps and those measured from reef segments (p = 0.019) with a degree of correlation (R^2^ = 0.5172) similar to those for DIC and NH_4_^+^ (Fig 3F). The regression coefficient for this relationship was significantly lower than the expected slope of one (*t* = 3.85, p = 0.004). Similar to denitrification, incubations of clumps alone overestimated SRP fluxes at the lower end and underestimated fluxes at the higher end of reef segments. SRP fluxes were not significantly correlated with oyster biomass (p = 0.45, R^2^ = 0.07; Fig 4F).

## Discussion

The majority of fluxes measured from clumps fell within the range of values measured for reef segments, which supports that much of the metabolism occurs within the clumps rather than in underlying sediments. While it is clear that removing clumps from the reef surface resulted in experimental artifacts, it is expected that methods that incubate sediments adjacent to oyster reefs would likely miss a proportion of nutrient and gas fluxes occurring without the oyster community. A number of efforts have pointed to the oyster as a potential denitrification hotspot; yet, direct comparisons of whole reef communities and their associated organisms to oyster clumps and associated organisms alone have not been made. The addition of cleaned bivalves to sediment without bivalves may result in less efficient denitrification than an acclimated reef [23] or rates that cannot be extrapolated to entire systems because they are not consistent with natural conditions. Our approach takes the importance of the oysters, associated-macrofauna, and shell microbial communities into consideration by leaving these components as undisturbed as possible. The present study builds on previous findings that oysters are associated with with high denitrification rates by providing a direct measure of how oyster clumps compare to an acclimated reef segment.

Overall, fluxes of DIC, NH_4_^+^_,_ and SRP measured from oysters alone were correlated with reef segment fluxes. During the trial run in June, we were concerned that the removal and placement of oyster clumps in new trays would result in dislodged labile organic matter and sediment from the shells. Nonetheless, the regressions of concentrations versus time to calculate fluxes were linear and significant for all nutrients, except for NO_2/3_^-^, indicating that for the most part, the incubation of clumps did not dramatically increase variation in biogeochemical processes within the time course of experiments. Our results provide evidence that oysters support much of the metabolism on the reef; however, it is worth noting that none of the clump fluxes met all of the criteria required for an accurate estimate of reef segment fluxes, such as highly correlated (R^2^≥ 0.80) variables, correlation coefficients that did not differ significantly from one, and intercepts that did not differ significantly from zero.

Although both measurement approaches were correlated for O_2_ and DIC-based respiration rates, oyster clumps overestimated O_2_ fluxes at the higher end of reef segments rates. The increase in O_2_ demand was likely due to the reoxidation of reduced chemical species occurring on the newly exposed surface area on the bottom of the clumps, considering previous studies have observed the development of anoxic sediments and increases in reduced chemical species below oysters during warmer months [23, 41]. In contrast to O_2_ fluxes, DIC-based respiration from oysters alone were the only fluxes that met the criteria of having a slope that was not significantly different from one and an intercept not significantly different from zero, indicating that remineralization rates were minimally affected the by the removal and placement of clumps.

Regression of NH_4_^+^ fluxes demonstrated that incubation of clumps alone produces a consistent error in measurements across fluxes that results in an overestimate of reef segment fluxes. It was uncertain why we observed higher NH_4_^+^ fluxes when oysters were incubated alone (Fig 3) but these results may suggest that dissimilatory nitrate reduction of nitrate to ammonium (DNRA) increased. DNRA can be promoted when the availability of organic carbon increases, NO_3_^-^ concentrations are low, and free sulfide (H_2_S and S^2-^) concentrations in adhered sediment are high [42–44]. Another possible explanation is that incubation of oyster clumps alone removed much of the shell hash surface area that would support nitrification and the removal of NH_4_^+^. We speculate that DNRA was stimulated when oysters clumps were incubated alone by concentrations of reduced sulfides that were exposed on the bottom of the oyster clumps.

Even though N_2_ and NO_2/3_^-^ fluxes from clumps were not significantly correlated with fluxes from reef segments, N_2_ fluxes tended to increase with increasing reef segment rate while NO_2/3_^-^ showed the opposite trend. As noted above, the increase in NH_4_^+^ fluxes and decrease in NO_2/3_^-^ fluxes measured from oysters alone may have allowed denitrification rates from oysters alone to have a positive relationship with whole tray estimates. Alternatively, rates of nitrification could have been reduced in our oyster only incubation simply through loss of surface area. The bottom of the *ex situ* incubation trays contain a large amount of shell and shell hash with high surfaces which could support high rates of nitrification. Interestingly, the underlying shell and shell hash does not appear to support high mineralization rates given minimal loss in DIC flux when the oyster clumps are incubated separately.

Regression of SRP fluxes from the two measurement approaches found that clump fluxes underestimated higher rates, suggesting strong binding of P to oyster shell. In addition, high CO_2_:SRP or ΣN:SRP ratios suggest that remineralized P was retained.

Positive relationships between several fluxes and oyster tissue biomass within the clump samples provides further evidence that much of the biogeochemical activity in this system occurs within oyster clumps rather than in underlying sediments. Fluxes of both O_2_ and NH_4_^+^ showed a significant positive correlation with oyster biomass and fluxes of DIC and N_2_ showed marginally significant positive correlations. Similarly, work by Green et al. [45] has shown that *Crassostrea gigas* densities are positively correlated with CO_2_ and CH_4_ fluxes, while Smyth et al. [26] have reported that rates of denitrification measured adjacent to reefs were positively correlated with oyster (*C*. *virginica*) densities. Given the spatial limitations of this work, future research should assess whether the observed relationships hold up at other sites with different sediment characteristics and hydrological conditions. Although the results from this subset of work are only from one site and season, this research is in agreement with previous observations that oysters stimulate denitrification and other biogeochemical transformations in reef environments [19, 21, 24].

### Implications

**Fig 5:**
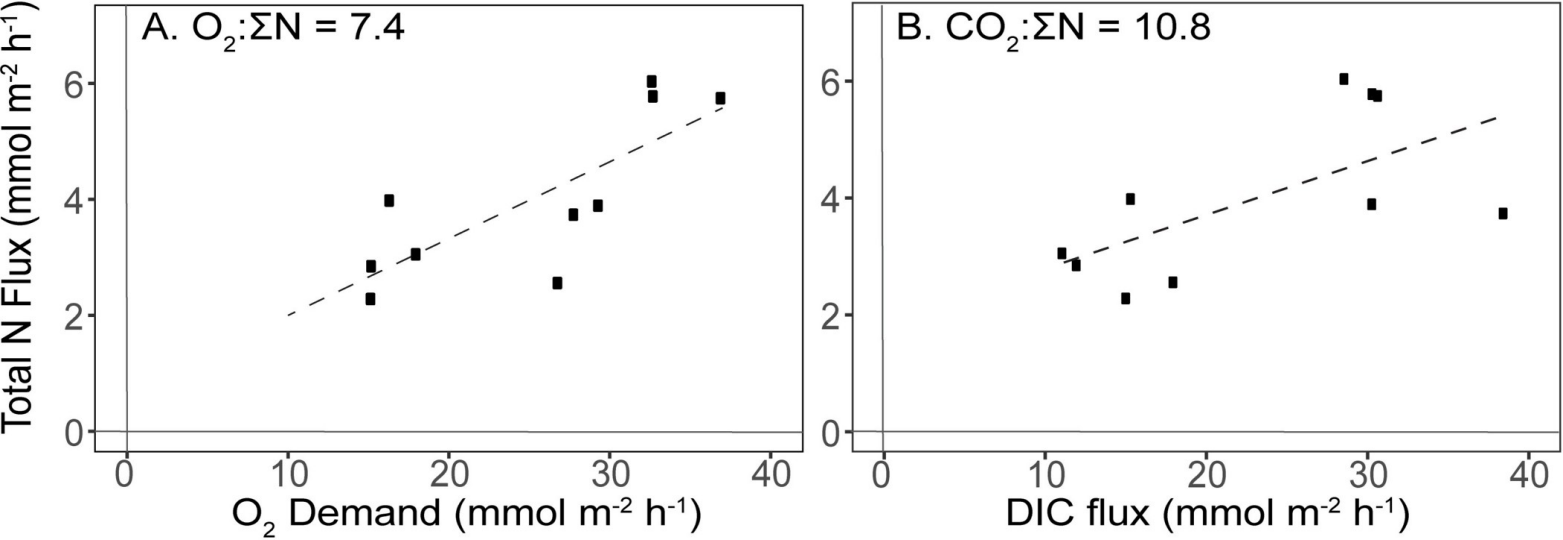
Stoichiometric plots using O_2_, DIC, and ΣN (sum of NH_4_^+^, NO_2/3_^-^, and N_2_-N) from individual fluxes of oysters alone. We assume that CO_2_:O_2_ is a 1:1 in the plot of (a) total N flux versus O_2_ demand and get a CO_2_:ΣN ratio of 7.4. This is compared to an actual plot of (b) total N flux versus DIC flux, which had a CO_2_:ΣN ratio of 10.8. Both ratios are similar to the Redfield elemental ratio for marine algae (106:16).

*In situ* approaches for measuring oyster denitrification and other biogeochemical fluxes are logistically challenging [20, 28], but incubations must include oysters if the goal of the research is to accurately quantify oyster reef biogeochemical fluxes. The observation that the incubations of oysters alone generally fall within the range of measured reef segment rates suggest that oyster clumps are driving reef-associated biogeochemistry. Similarly, we were able to confirm that oysters alone account for the majority of the nitrogen transformations, with stoichiometric plots consistent with the expected Redfield ratio for marine algae (Fig 5; [19]). Nitrification supported by oyster shell surface area is likely key to maintaining high rates of denitrification in our study. In addition, despite the potential for production or dissolution of carbonates [46], this work suggests that the use of DIC, instead of oxygen may be preferred for determining total nitrogen transformations considering DIC fluxes were consistent between treatments. From these experiments, estimates of nitrogen removal as an ecosystem service are best characterized by including the whole community: sediments, oysters, and the oyster-associated metazoan community. We recognize that whole community incubations are considerably more difficult than incubations of oysters or sediments; however, other approaches will necessarily result in low estimates of denitrification. This study highlights the role that oysters play in nutrient transformations and the importance of including intact segments of oyster reefs in incubations for a complete assessment of denitrification and other biogeochemical fluxes.

The data herein point to an important consideration in the determination of whether oyster-associated N removal should be a BMP for overcoming eutrophication. Much of the data available for BMP consideration focuses on the role that oysters play in stimulating denitrification in the surrounding sediments, rather than within the reef and associated organisms. As demonstrated by the high rates of N transformations from clumps of oysters alone, the coupled processes occurring on oyster reefs are complex, and require an improved understanding of the environmental drivers stimulating oyster-associated denitrification to establish oysters as a BMP.
